# Engaging rural communities in Bangladesh to tackle antimicrobial resistance through the Community Dialogue Approach: a process evaluation protocol for COSTAR project in Cumilla, Bangladesh

**DOI:** 10.3389/fpubh.2024.1466780

**Published:** 2024-12-13

**Authors:** Md Badruddin Saify, Nichola Jones, Fariza Fieroze, Jessica Mitchell, Joseph Paul Hicks, Samina Huque, Sajib Saha, Sophia Latham, Helen Hawkings, Rumana Huque, Rebecca King

**Affiliations:** ^1^ARK Foundation, Dhaka, Bangladesh; ^2^Nuffield Centre for International Health and Development, Leeds Institute for Health Sciences, University of Leeds, Leeds, United Kingdom; ^3^The Global Academy of Agriculture and Food Systems, The Royal (Dick) School of Veterinary Studies and The Roslin Institute, University of Edinburgh, Edinburgh, United Kingdom; ^4^Department of Livestock and One Health, Institute of Infection, Veterinary, and Ecological Sciences, University of Liverpool, Neston, United Kingdom; ^5^Malaria Consortium, London, United Kingdom; ^6^Department of Economics, University of Dhaka, Dhaka, Bangladesh

**Keywords:** antimicrobial resistance, AMR, community engagement, Community Dialog Approach, Bangladesh, process evaluation, complex intervention

## Abstract

**Introduction:**

Antimicrobial resistance (AMR) is a global problem and is especially threatening for low-and-middle income countries like Bangladesh. The COSTAR (Community-led Solutions to Antimicrobial Resistance) project includes a Randomised Control Trial (RCT) which aims to evaluate the effectiveness of the Community Dialog Approach (CDA) to improve levels of correct and appropriate knowledge and reported practice about antibiotics, antibiotic use, and antibiotic resistance (ABR) from a One Health perspective, among adult community members in 5 selected sub-districts of Cumilla. The CDA is a community engagement approach involving community members in active discussions also known as Community Dialogs (CD), run by local facilitators. The dialogs promote collective action to produce sustainable social change. The trial’s process evaluation will evaluate fidelity, dose, adaptation, reach, mechanisms of impact and the process of knowledge diffusion using the MRC framework for the evaluation of complex interventions.

**Methods and analysis:**

The process evaluation will be implemented in the catchment areas of 25 selected community clinics (CCs) in the intervention group. The key actors involved in the process evaluation are participants from master trainers and trainers training; community dialog facilitators; supervisors; community dialog participants and non-participants; and local and national level government stakeholders. Qualitative and quantitative data will be collected through Focus Group Discussion (FGDs); Case Studies; Key Informant Interview (KIIs); CD observations; monitoring forms; quarterly feedback from facilitators and supervisors, and pre-and-post-test questionnaires administered during the training of facilitators. All qualitative data will be coded using *a priori* coding framework in NVIVO 14. Quantitative data will be analysed using descriptive statistics.

**Ethics and dissemination:**

Ethical approval was obtained from the Bangladesh Medical Research Council (BMRC): BMRC/NREC/2019–2022/427 and from the University of Leeds Faculty of Medicine and Health ethics board: MREC 20–034. All results will be disseminated through a one pager summary; infographics; peer-reviewed journal articles and national and international conferences.

**Clinical trial registration:**

https://www.isrctn.com/ISRCTN93756764, identifier ISRCTN93756764.

## Introduction

Antimicrobial Resistance (AMR) is one of the biggest major threats to global health and well-being; responsible for approximately 1.27 million human deaths in 2019 alone (1, 2). A true One Health issue (3), AMR stands to impact not only human health outcomes but also the health of animals and the environment, particularly placing food security andsocio-economic development at risk (4).

Addressing AMR requires multi-sectoral action across all One Health sectors and beyond; development, humanitarian agencies and policy makers are important actors in reducing the impacts and scale of AMR (5).

Community Engagement (CE) is considered to be an effective method to address individual and community level health issues (6, 7). CE can, when incorporated into research design, generate equitable partnerships with communities, promoting knowledge exchange and co-production of locally appropriate solutions to complex health issues (8). An understanding of the drivers of AMR in Bangladesh, as well as any barriers to sustainable change are essential to making progress toward AMR reduction. Community voice is, therefore, an essential component to any intervention aiming to address AMR. Indeed, other studies demonstrate a need to go beyond traditional engagement approaches to increase awareness in order to tackle AMR in low resource settings and CE provides an effective approach to achieve this (6–10). Effective CE strategies can induce positive transformations in the knowledge and behavior of community members, empowering them to engage in policy-level discussions and interact with stakeholders to whom they typically lack access, thereby enabling them to address AMR in low-resource settings (11). Our previous pilot project showed promising results for scaling up a CE method called Community Dialog Approach (CDA) in Bangladesh to tackle AMR (12). The CDA was developed by Malaria Consortium and is based on the Integrated Model of Communication for Social Change (IMCFSC) (13, 14). The CDA engages community members in active discussion sessions also known as Community Dialogs (CD), run by local facilitators, and promotes collective action to produce sustainable social change.

### Community solutions to antimicrobial resistance (COSTAR)

The ‘COSTAR’ project aims to address the drivers of AMR in community settings in Bangladesh and Nepal. In Bangladesh, the CDA was implemented across the catchment areas of 25 community clinics (CCs), each of which has an approximate population of 6,000 and there are additional 25 CCs areas as control sites whose population did not receive any CDA. This approach is being evaluated through a cluster-randomized control trial (RCT) to assess the effectiveness of CDA for improving knowledge, attitudes and reported practices in relation to the drivers of AMR and a qualitative process evaluation to understand the implementation, mechanisms of impact and potential outcomes of the project. The full description of interventions is reported in trial protocol (forthcoming). A summary of the intervention is presented below

The CDA takes a four-phase approach, preparation; pre-implementation; implementation and evaluation. In the preparation phase, the researchers developed a research protocol, prepared a theory of change document and drafted the overall study design based on the learnings from previous studies in Bangladesh and in Nepal (12, 15, 16). Researchers in the ARK Foundation (17) (Bangladesh Implementation Partner) also conducted multiple sensitization meetings with national and local stakeholders to discuss the proposed intervention to ensure it is contextually appropriate. In pre-implementation, researchers co-produced intervention toolkit (a flipbook, a dialog facilitation guide, monitoring forms-decision log, CD report and supervision meeting checklist). Three-layers of training (master trainer training, training of trainers and training of facilitators and supervisors) were organized to ensure that the CDA process is embedded into the national and local healthcare infrastructure and has the potential for future scale-up. Local community volunteers (herein referred to as ‘Community Dialog Facilitator-CDF’) from the community clinic (CC) catchment villages were recruited and supervised by either the community health care provider (CHCP) from the CC that covers the community in which they will deliver and facilitate the CDs, or by their local health assistant (HA) or assistant health inspector (AHI). The supervisors of the community dialog facilitators were first identified with the help of local officials from Ministry of Health and Family Welfare (MoHFW) attached at Upazilla Health Complex (UHC) from each sub-district. Two Supervisors, Community CHCP and HA or AHI were selected from each cluster area. The supervisors were invited to participate in a sensitization meeting in their own sub-district and pre-fixed selection criteria for CDF were explained to them. They were provided with selection forms to nominate and recruit CDF from villages in their own cluster. The selection criteria stated that CDFs must be ≥18 and at least complete secondary school certificate exam (SSC); good relationship with their community members and have the motivation to engage community members in discussions through CDA. Depending on the size of each village either one or two pairs of CDF (each pair consisting of one male and one female candidate) were recruited.

CDFs and supervisors participated in three-day long training of facilitators and supervisors in their own clusters. Training was provided by local government personnel (herein referred to as Trainers) attached to UHC and/or Upazilla Livestock Office. These trainers were first trained by master trainers in training of trainers. Master trainers were a group of national specialists on AMR and One Health attached to MoHFW, Ministry of Livestock and Fisheries (MoLF) and national members of the research team. The master trainers were also trained in the master trainer training by the international specialists on CDA from Malaria Consortium (18).

In the implementation phase, CDF were provided with a toolkit to implement the intervention consisting of a flipbook, discussion guide and scorebook. Facilitators were asked to use this toolkit to conduct 11 CD sessions on 11 distinct topics, repeated across 2 different venues within their respective villages. The flipbook was divided according to the 11 distinct topics, with each topic addressed in one session. To aid facilitators in sharing key points for each topic area, the flipbook was designed to stand upright; a contextually appropriate illustrated image on each page faced the community while the reverse side had written (in Bangla) prompts for facilitators on key messages. The community dialogs included topics including matching diseases and microbes with the right kind of medication; access to, and safe utilization of antimicrobials; AMR: Human and Animal Health Promotion; Animal and Fish Care and Taking a One Health Approach.

More detailed description of the selection and development of topics and messages can be found here (11):. The discussion guide was prepared to assist the CDF preparing their CD sessions and a scorebook was provided to keep the records of participant’s opinions on each CD sessions. The overall delivery phase of the project was 12 months. However, as Bangladesh is a Muslim majority country, the holy month of Ramadan was excluded from the delivery phase after extensive discussion between the research team, local and national level stakeholders.

In each CD, the CDF is responsible for maintaining two separate forms; decision logs and CD Reports. The decision log is a two-page document which records the key decisions taken by the community members during the CD and allows CDFs to follow up on those decisions and resulting actions in the next CD at the same venue. Responses on implementing or following decisions will be recorded using three level of responses (‘Met’, ‘Partially met’ and ‘Not met’). The CD Report is a two-page document outlining the time, date, duration, location, number of participants in CD sessions (gender distribution) and key challenges faced, if any. The CDF is required to fill up one decision log and one CD report for each CD they organized. CDFs also attended a monthly supervisory meeting conducted by their respective supervisors in the local community clinic (CC). They shared the planning for organising CDs in their respective villages in each month using a monthly planning form and discussed any issues or challenges relating to facilitating CDs. Supervisors are responsible for completing one supervision meeting checklist for each facilitator they supervise each month. All facilitators and their supervisors received quarterly refresher training sessions embedded in the quarterly feedback meeting. ARK Foundation conducted this training and feedback meeting for one day in each sub-district to refresh CD contents, address any issues faced by facilitators, and to collect monitoring forms. Four quarterly feedback meeting and refresher training were organized in each sub-districts across 12 months of intervention period. Details on the monitoring forms are discussed in Data Collection section.

### Process evaluation

**Figure 1:**
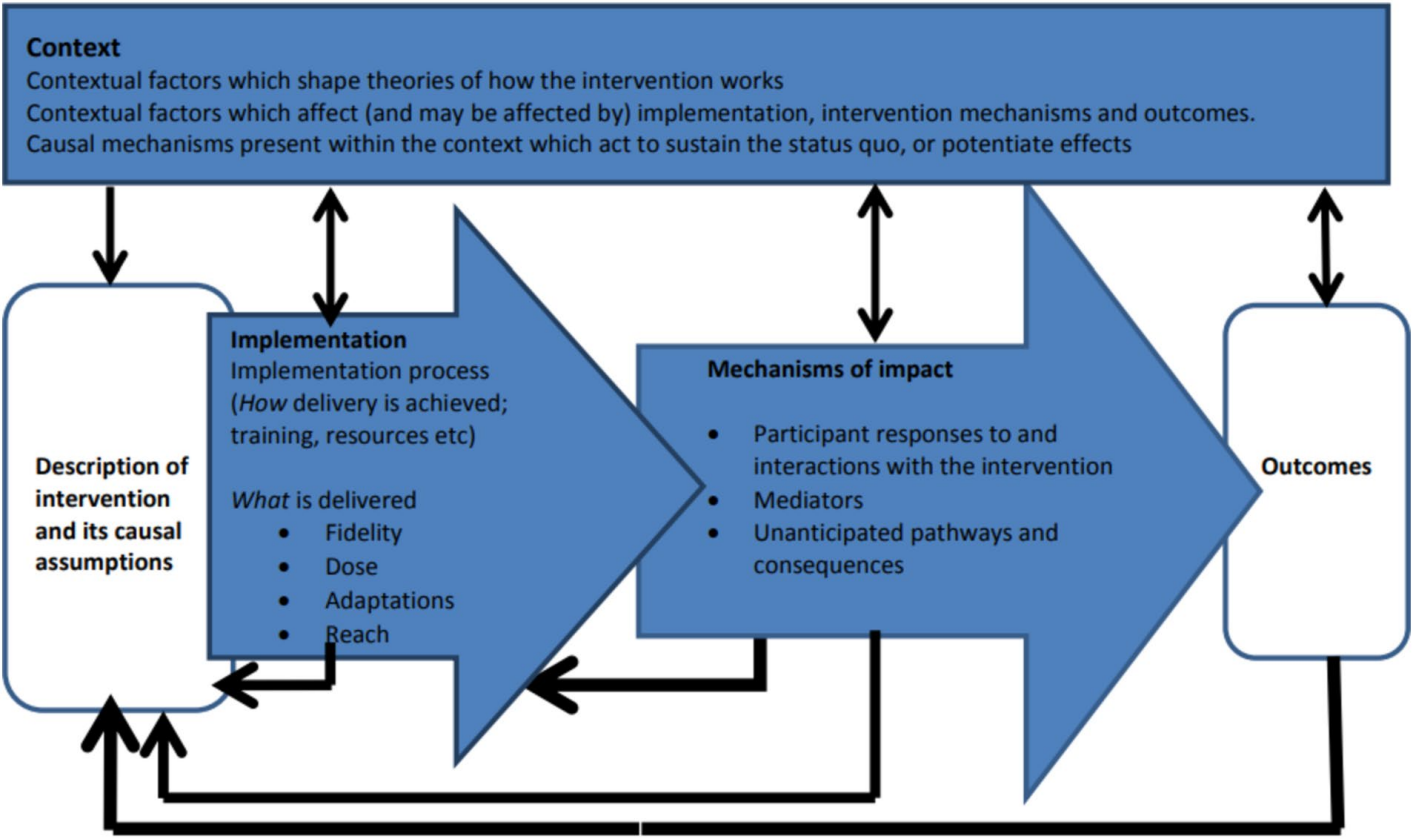
MRC process evaluation framework for complex intervention.

While the RCT aims to evaluate whether the CDA intervention can improve levels of correct and appropriate knowledge and reported practice about antibiotics, antibiotic use, and antibiotic resistance (ABR) from a One Health perspective, among adult community members within the study settings, we also aim to evaluate the process of intervention guided by the Medical Research Council (MRC) framework for complex interventions (Figure-1) (19). Complex interventions are usually described as interventions that contain several interacting components, requiring tailored approaches to suit the needs of the health issue and setting (20).

We seek to answer the following questions through our process evaluation: (1) How the master trainers, trainers, facilitators and supervisors experience the training on CDA and how participatory the training was?; (2) How the facilitators, supervisors, local community members and local key stakeholders experience the CDA and to what extent the CDA is embedded in the existing healthcare infrastructure? (3) How the CDA impacts on people’s lives and their behavior in relation to antimicrobial resistance and antimicrobial consumption? Our overall aim is to assess how CDA as an intervention is implemented, experienced, and embedded within community and healthcare settings to promote sustainable knowledge and behavioral changes regarding antibiotic use and resistance.

The objectives of our process evaluation are:

- The extent to which the intervention was implemented as intended (looking at fidelity, dose, adaptations and reach)
- The mechanisms of impact (participant responses to and interactions with the intervention)
- To explore processes through which new knowledge, knowledge diffusion, practices and policy change may be emerging and spreading within community settings.

## Methods and analysis

A primary mixed methods study will assess our intervention’s process. Qualitative data from key points across the intervention will be gathered, collated and analysed to facilitate a process evaluation of the CDA implementation in the Cumilla District of Bangladesh. Quantitative data will come from community dialog observations, pre/post-tests, and monitoring forms.

We will examine and assess data from CD observation checklists, decisions logs, CD reports and other sources to understand the implementation process (fidelity, adaptation, reach, dose) and mechanism of impact of the intervention.

### Study settings

In Bangladesh, we scaled up the intervention into rural settings of five sub-districts (Daudkandi, Homna, Brahmanpara, Burichang and Barura) in Cumilla. Cumilla is located around 100 km south-east of the capital Dhaka and, according to the 2022 census, has a population of 6,212,216 million living in 17 sub-districts. The five sub-districts of our study are home to an estimated 1,672,505 people. From each sub-district, 10 Community Clinics (CCs) were randomly selected, where intervention to control ratio is 1:1. Each community clinic area is defined as a cluster. CCs are government-run, community health facilities providing basic healthcare and situated at the last tier of the healthcare infrastructure in the country. The intervention was conducted on 25 CCs with 1–6 villages under their catchment areas, with each CC having a different number of villages.

### Theoretical approach

**Figure 2:**
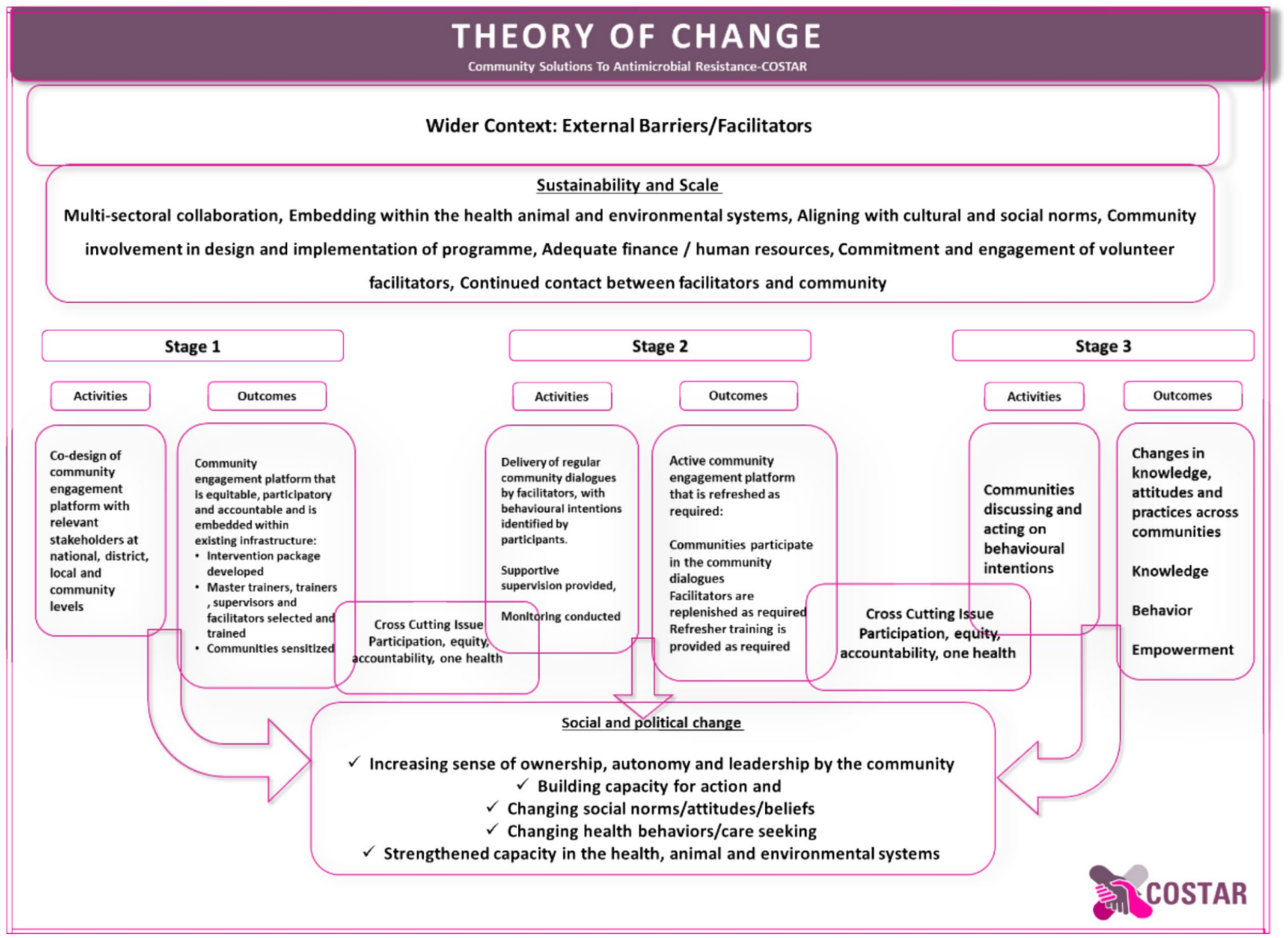
COSTAR theory of change.

Our process evaluation is designed based on the theory of change for COSTAR and provides the context for evaluation (Figure 2).

Our theory of change seeks to create meaningful outcomes through several strategic stages. During stage one, we prioritize co-designing a community engagement platform with stakeholders at the national, district, local, and community levels. Our goal is to build an equitable, participatory, and accountable platform that integrates with current infrastructure. We create an intervention package and carefully select and train master trainers, supervisors, and facilitators, all while raising community awareness about the value of our efforts. Moving on to stage two, we will focus on facilitating regular community discussions using our trained facilitators, ensuring that facilitators’ behavioral intentions are reinforced through supervision and monitoring. This results in an active community involvement platform that changes as needed, including continuing conversation participation, facilitator replenishment, and refresher training. Finally, in the third stage, we empower communities to discuss and act on their behavioral intentions, resulting in actual changes in knowledge, attitudes, and behaviors across communities. We work to promote social and political change by instilling a sense of ownership, autonomy, and leadership in the community, as well as increasing action capacity and transforming social norms, attitudes, and beliefs. Finally, our method helps to build capacity in health, animal, and environmental systems of the government embedding into existing infrastructure, guided by the principles of participation, equity, accountability, and one health.

### Study participants

We will include a diverse range of respondents for this study. The list of respondents will include the trainers involved in three layers of training, CDF and their supervisors from each sub-district, participants and non-participants who did and did not join in the CD sessions in their community from each sub-districts, key local community stakeholders, local and national subject and topic experts on AMR and One Health. We will approach and select Community Dialog Facilitators (CDF), Supervisors, CD Participants, CD non-participants and local community stakeholders based on some pre-fixed selection criteria. CD participants, non-participants and the local community stakeholders will be approached by the research team with the help of CD facilitators and Supervisors from respective intervention areas.

We will select the study participants from 25 intervention areas. All the samples will be purposively selected to capture the diverse characteristics of study participants.

The research team of ARK Foundation will contact details of participants is illustrated in Table 1.

**Table 1:** Study participants in process evaluation of COSTAR.

Study participants	Approx. No. of participants	Operational definition
Participants from Training of Master Trainers (ToMT)	~6	Central level government officials who are expert on AMR and One Health from MoHFW and Department of Livestock (DLS) under the Ministry of Fisheries and Livestock (MoFL) who participated in the Training of Master Trainers and subsequently provided training to trainers.
Participants from Training of Trainers (ToT)	~8–12	Local level government officials from MoHFW and Department of Livestock (DLS) under the MoFL who participated in the Training of Trainers (ToT) and subsequently provided training to the Training of Facilitators and Supervisors (ToFS).
Community Dialog Facilitators (CDF)	~50–60	Local community members from intervention areas who participated in the Training of Facilitators (ToFS) and facilitated Community Dialogues (CD) within their sub-districts will be involved in the study. Both regular and irregular Community Dialog Facilitators (CDFs) will be invited to participate in the process evaluation.
CD supervisors	~20	Community Healthcare Providers (CHCPs), Health Assistants (HAs), and Assistant Health Inspectors (AHIs), who served as supervisors for each intervention implementation area, will also be included.
CD participants	~140–150	Local community members, including men, women, and individuals with additional needs, who attended the community dialogues in their villages will be invited to participate in the process evaluation. Invitations will be extended based on the number of CD sessions attended, ensuring both low and high participation levels are represented.
CD non-participants	~20–30	Local community members, including men, women, and individuals with additional needs, who did not attend the community dialogues conducted in their villages.
Local community stakeholders	~50–60	Local community people across One Health sectors from intervention areas. Local community stakeholders could be: - Union Parishad member - Religious leader (Imam/Purohit) - Teacher - Influential Person from the community - Animal health stakeholders - Small/Large animal/crop farmers - Social Worker - Health Worker affiliated with NGO or government.
Local and national level policymakers and AMR and one health stakeholders	~10	We will approach to local level policy makers such as Upazilla Health and Family Planning Officer (UHFPO) from Upazilla Health complex (UHC). We will also approach to National level policy makers from Communicable Disease Control (CDC) of Directorate General of Health Services (DGHS) and Directorate General of Drug Administration (DGDA) under MoHFW, Academicians, Researchers and NGO stakeholders associated with AMR and One Health spectrum to understand the policy implication of community engagement and CDA to tackle AMR in the community from One Health perspective.

### Data collection

We will employ different qualitative approaches to understand different aspects of intervention implementation. We will collect data from February 2024–August 2024. Core research team members experienced in qualitative data collection methods will conduct KIIs, Case Studies and FGDs using the pre-developed topic guides. Topic guides will cover broad themes linked to the theory of change of this project to explore the research objectives. The broad themes are: Co design; Implementation; Behavior Change; Mechanism of Impact; Sustainability and Scale; Cross-Cutting issues and Capacity Development. All data will be collected in Bangla language which is the primary language of Bangladesh and those communities.

We will approach the identified respondents and explain the objective of our study, why we decided to reach out to them, and the potential benefits and risks involved. We will explain the information sheet underlining the study’s details and objectives, their right to withdraw, data sharing and benefits and risks involved associated with this study. We will provide participants enough time to read through the information sheet provided and ask any questions if they do not understand anything. We will provide a copy of information sheet and consent forms to each respondent. Once the respondents understand the study objectives and decide to participate in the study, we take written consent from them and if any participant is unable to write or sign his/her own name, we will take thumb print along with a witness’s signature. We will proceed with FGDs and interviews with participant’s explicit written consent. The discussions and interview will take place at a location and time determined by the participants, protecting their anonymity and privacy. To further respect participants’ preferences, male and female interviewers/facilitators will conduct interviews or focus group discussions with members of their respective genders. Each interview will last between 45 and 60 min, with FGDs lasting 60 to 90 min. We will conduct the interviews and discussions face-to-face. However, if any group of participants decide to conduct the discussion or interviews through cloud-based meeting software, we will respect their wishes and hold the discussion and interviews through cloud-based meeting software. We will send an electronic copy of information sheet and written consent to the participants before the discussion or the interviews and obtain their written consent.

Responses from FGDs, KIIs and case studies will be audio-recorded.

### FGDs

Focus group discussions (FGDs) will be conducted with respondents of Master trainer training, Training of Trainers, CDF, CD Supervisors, CD Participants and Non-participants and Local stakeholders. The details of participants and number of FGDs are illustrated in Table 2.

**Table 2:** Details of process evaluation data collection and sampling strategy.

Study participants	Data Collection Methods	Number of Samples	Selection Criteria	
Participants from training of master trainers (ToMT)	Focus Group Discussion (FGD)	1 FGD	We will invite all the participants from training of master trainers (ToMT) to take part in the FGDs.	
Participants from training of trainers (ToT)	Focus Group Discussion (FGD)	1 FGD with Trainers	We will invite the engaged and available trainers from ToT to participate in FGD.	
CD facilitators	Focus group discussion (FGD) Case studies	1 FGD in each sub-districts, in total 5 FGDs. 2 case studies; one with male and one with female facilitators	For FGDs: We will first divide the CD facilitators in three categories. We will include and invite the same number of male and female CD facilitators for each FGDs. In each FGD there will be mixture of respondents from each group. Definition of the categories are presented below:	
			*- Group-1*	Facilitators who conducted 20–22 CD sessions Whose CD participants average was 20–25 Attended all quarterly feedback meeting Illustrated good performance during CD observation by research team
			*- Group-2*	Facilitators who conducted 14–20 CD sessions Whose CD participants average was more than 15–20 Attended all quarterly feedback meeting Illustrated Moderate to good performance during CD Observation by research team
			*- Group-3*	Facilitators who conducted less than 14 CD sessions Whose CD participants average was less than 15 participants Attended Less than 3 quarterly feedback meeting Illustrated below average performance during CD Observation by research team
			For case studies: We will purposively select one male and one female facilitators for case studies based on their overall CD performance and responses during the FGDs on reported behavior change and experiences related to intervention implementation.	
CD supervisors	Focus group discussion (FGD)	2 FGDs	We will categorize the supervisors in two categories; active and partially active. The definition of the categories is presented below. The number of female supervisors are large in numbers, therefore, we cannot ensure to include equal number of male and female supervisors in FGDs.	
			Active	1. Supervisors whose CD facilitator dropdown rate was less 2. Supervisors who regularly held Supervision meeting 3. Supervisors who monitor his/her Facilitators regularly
			Partially active	1. Supervisors whose CD facilitator dropdown rate was comparatively higher 2. Supervisors who are less regular to hold supervision meeting 3. Supervisors who partially monitor his/her facilitators
			We will obtain the information required to categorize the supervisors from the supervision checklist and report form as well as the field research team observations. We will purposively select the supervisors from two categories. We will try to include equal number of respondents from each categories.	
CD participants	Focus group discussion (FGD) Case studies	2 FGDs in each sub-districts, One with Male CD Participants and One with Female CD Participants; in total 10 FGDs. 3–4 Case studies; one with male and one with female Participants and 2 with people with additional needs	For FGDs: We will invite CD participants (male and female) from each intervention areas (CCs) who attended CDs in their areas regularly. We will take assistance from our CD facilitators and supervisors in identifying and inviting participants from their local areas. For case studies: We will purposively select one male and one female CD participants based on the reported behaviour and knowledge change. We will take assistance from our CD facilitators and supervisors in identifying such CD participants and the participants with additional needs.	
CD non-participants	Focus group discussion (FGD) Case study	2 FGDs 1 Case study with non-participants with additional needs	We will invite CD non-participants from each intervention areas (CCs) who never attended CDs in their areas. We will take assistance from our CD facilitators and supervisors in identifying and inviting non-participants from their local areas.	
Local community stakeholders	Focus group discussion (FGD)	1 FGD in each sub-districts, in total 5 FGDs.	We will invite local community stakeholders from each sub-districts. Our field research team first identify the respondents from intervention areas and will prepare a list of them to invite in FGD.	
Local and national level policymakers and AMR and one health stakeholders	Key informant interviews (KIIs)	5 KIIs.	We will conduct 1 KII with local UHFPO from one sub-district, two KIIs from MoHFW, 3 KIIs with academicians, researchers and NGO stakeholders.	

### KIIs

Key Informant Interviews (KII) will be conducted with Local level stakeholders involved human or animal health, representatives from Communicable Disease Control (CDC) of Directorate General of Health Services (DGHS) and Directorate General of Drug Administration (DGDA), academicians and NGO professionals working in tackling AMR and in One Health. The key reason to select representatives from CDC and DGDA is because they are the key stakeholders in tackling AMR in the country, while CDC host the national Antimicrobial Resistance Containment (ARC) program and DGDA is responsible for regulating pharmacies and implementing Acts and legislation in pharmacies. The details of participants are illustrated in Table 2.

### Case studies

We are planning to conduct case studies through in-depth interview with CDF, CD participants and non-participants. We will also aim to interview from CD participants and non-participants with additional needs to ensure inclusivity. Both male and female CDF and participants/non-participants will be interviewed to ensure gender responsiveness (Table 2).

### Community dialog observation

The research team have observed 30 CD sessions using a pre-prepared checklist, 6 CD sessions from each sub-district. Two team-members of the research team observed each CD session and record the process of CD session in the checklist. This checklist consists of both quantitative and qualitative responses (Table 3).

**Table 3:** Details of quantitative data.

Data source	Variable types	Response type	Data collection method
CD observation	Venue accessibility	Yes/No	Observation by independent researcher
	Participants number	Number	
	Responsiveness of CD participants (gender disaggregated)	Yes/No	
	Following of CD steps	Yes/No	
CD report	Duration of CD	Number	Self-reported
	Type of CD Venue	Categorical numbered response	
	Number of CD participants (gender disaggregated)	Number	
Pre-test/post-test	General knowledge on microbes, correct behavior around antimicrobial usage and antimicrobial resistance	Yes/No	Self-reported

### Feedback from CDF in quarterly feedback meeting

In each quarterly feedback meeting, a different set of questions were administered to gather feedback from the CDF and their supervisors. A team member of the research team first explained each question to CDF and supervisors. The CDF and supervisors were divided into different groups according to their cluster (CC). Each group were then provided with same questions to provide their response. All CDF and their supervisors were asked to write down their experiences and responses for the questions in the provided paper.

### Monitoring tools

The CDFs have used and completed two monitoring tools (decision logs and CD reports) to capture the collective decisions from community and to report each of their completed CD sessions. We have collected those forms from CDF during each quarterly feedback meeting for further analysis. These forms contain both quantitative (e.g., time, date, venue and gender disaggregated number of participants) and qualitative data (e.g., collective decisions from the community) (Table 3).

### Pre and post-test data

A pre and post-test questionnaire was administered during the training of facilitators and supervisors and refresher training in each quarter to capture their understanding of the training contents before and after the training. The respondents age, sex and educational level were also collected (Table 3).

## Analysis

Transcripts will be generated from each audio recordings. All transcripts will be translated from Bangla to English. All personal identifiable information (PII) will be discarded, and all transcripts will be anonymised following UK Data Service anonymisation guideline for qualitative data (21). All data analysis will be conducted in English language. The study team from study site (Bangladesh) and UK will meet and collaborate using online meeting platform periodically.

### FGDs, KIIS, case studies and feedback from CDF in quarterly feedback meeting

We will conduct a thematic framework analysis, concentrating on key implementation outcomes such as fidelity, dose, adaptation, reach and mechanism of impact with gender and one health aspect incorporated as cross-cutting variables.

Our analytical approach will be systematic and iterative, combining deductive and inductive methodologies to examine interview, case study and FGD data. After obtaining all transcripts, our research team members will carefully read and reread them to become acquainted with the data. Before we begin coding, we will first create *a priori* coding framework. This coding framework will be developed based on the topic guides and transcripts obtained. An iterative process will be followed by the research team to finalize the framework.

To ensure consistency and intercoder reliability, separate coders will initially code the same transcripts with the produced codebook. We will use the NVIVO 14 Pro software for effective coding. The coding framework will be formatted within NVIVO 14 to ease the coding process.

#### Community Dialog observation

Data from Community Dialog observations will be collated into an MS Excel spreadsheet. Quantitative and qualitative data will be recorded in two separate spreadsheets. Qualitative data will be further analysed using inductive approach.

#### Monitoring tools

Data from decision log and CD report will be collated into an IBM SPSS Statistics 20 database. All the qualitative data such as decisions from the decision logs will be coded using a pre-prepared codebook to categorize the decisions. Quantitative data will be analyzed using simple descriptive statistics and qualitative data will be analyzed using thematic framework analysis.

#### Pre and post test data

Pre and post test data will be collated in an IBM SPSS Statistics 20 database. To understand the effectiveness of the training contents, we will perform simple descriptive statistical analysis to check the knowledge level of training participants before and after the training.

### Patient and public involvement

Our process evaluation is robust and comprehensive as we plan to involve all relevant stakeholders from all sectors involved. The study participants, government and non-government actors and the public from the study area will also be interviewed to understand their perceptions on the implemented intervention to tackle AMR in the community and the feasibility of the intervention in the local context and the possibility of embedding the CDA approach within existing healthcare infrastructure. The results of the process evaluation will be shared with the local community stakeholders where the intervention is implemented and with the national level stakeholders to better inform the policy change through dissemination meeting and providing one-pager easy to understand summary and infographics in Bangla language. In the final manuscript, all the study participants and the community members will be acknowledged for their contributions.

### Ethics and dissemination

Findings from the study will be disseminated through dissemination meeting, peer- reviewed publication, national and international conferences and through stakeholder consultation meetings.

## Conclusion

Antimicrobial resistance cannot be tackled without active participation from community members. Tackling AMR requires active and engaging solutions in low-resource settings and we believe that CDA can play a crucial role in tackling AMR in rural Bangladeshi communities. We are expecting that our process evaluation findings will inform us about the contextual factors of our intervention and the challenges to implement it within the existing healthcare structure in Bangladesh. If we success with this intervention, we can inform the policymakers to scale-up and embed this approach within the human and animal healthcare structure in low-resource settings in the country. If we fail, the process evaluation will help us to understand the reasons behind the implementation or intervention failure. We will use the findings and lessons learned from this study to carefully design future interventions that are more tailored to the specific needs and constraints of the healthcare system in Bangladesh.
